# Web monitoring of emerging animal infectious diseases integrated in the French Animal Health Epidemic Intelligence System

**DOI:** 10.1371/journal.pone.0199960

**Published:** 2018-08-03

**Authors:** Elena Arsevska, Sarah Valentin, Julien Rabatel, Jocelyn de Goër de Hervé, Sylvain Falala, Renaud Lancelot, Mathieu Roche

**Affiliations:** 1 Unit for Animals, Health, Territories, Risks and Ecosystems (UMR ASTRE), French Agricultural Research for Development (CIRAD), French National Institute for Agricultural Research (INRA), Montpellier, France; 2 Institute of Infection and Global Health (IGH), School of Veterinary Science, University of Liverpool, Liverpool, United Kingdom; 3 Unit for Land, Environment, Remote Sensing and Spatial Information (UMR TETIS), French Agricultural Research for Development (CIRAD), Montpellier, France; 4 LabEx NUMEV, Laboratory of Informatics, Robotics and Microelectronics (LIRMM), University of Montpellier, French National Center for Scientific Research (CNRS), Montpellier, France; 5 Unit for Animal Epidemiology (UMR EPIA), French National Institute for Agricultural Research (INRA), Clermont-Ferrand, France; 6 University of Montpellier, Paris Institute of Technology for Life, Food and Environmental Sciences (AgroParisTech), French Agricultural Research for Development (CIRAD), French National Center for Scientific Research (CNRS), National research Institute of Science and Technology for Environment and Agriculture (IRSTEA), Montpellier, France; Swedish National Veterinary Institute, SWEDEN

## Abstract

Since 2013, the French Animal Health Epidemic Intelligence System (in French: Veille Sanitaire Internationale, VSI) has been monitoring signals of the emergence of new and exotic animal infectious diseases worldwide. Once detected, the VSI team verifies the signals and issues early warning reports to French animal health authorities when potential threats to France are detected. To improve detection of signals from online news sources, we designed the Platform for Automated extraction of Disease Information from the web (PADI-web). PADI-web automatically collects, processes and extracts English-language epidemiological information from Google News. The core component of PADI-web is a combined information extraction (IE) method founded on rule-based systems and data mining techniques. The IE approach allows extraction of key information on diseases, locations, dates, hosts and the number of cases mentioned in the news. We evaluated the combined method for IE on a dataset of 352 disease-related news reports mentioning the diseases involved, locations, dates, hosts and the number of cases. The combined method for IE accurately identified (F-score) 95% of the diseases and hosts, respectively, 85% of the number of cases, 83% of dates and 80% of locations from the disease-related news. We assessed the sensitivity of PADI-web to detect primary outbreaks of four emerging animal infectious diseases notifiable to the World Organisation for Animal Health (OIE). From January to June 2016, PADI-web detected signals for 64% of all primary outbreaks of African swine fever, 53% of avian influenza, 25% of bluetongue and 19% of foot-and-mouth disease. PADI-web timely detected primary outbreaks of avian influenza and foot-and-mouth disease in Asia, i.e. they were detected 8 and 3 days before immediate notification to OIE, respectively.

## Introduction

New and exotic animal infectious diseases are one of the major threats to global health security, especially regarding their zoonotic and pandemic potential, and economic impact on affected countries. One objective of proactive animal health authorities is therefore the implementation of epidemic intelligence activities, including early detection, verification and communication of signals of disease emergence from both formal and informal information sources [1].

Most animal health authorities have long-established formal, indicator-based surveillance, i.e. collection and reporting of quantitative indicators (e.g. number of cases) obtained from routine disease surveillance programs [1]. However, indicator-based surveillance has been shown to lack timeliness in the detection and reporting of exotic pathogen outbreaks. One example is the transcontinental spread of the African swine fever virus (ASF) from Southeast Africa into Eastern Europe in 2007 [2]. The first ASF cases were observed before May 2007 in Georgia, in the Caucasian region, but they were only officially confirmed in June 2007. ASF detection was based mainly on clinical findings, and only a small proportion of cases led to laboratory investigations, resulting in widespread infection among the porcine population of Eastern Europe [3].

The challenge facing most animal health authorities thus lies in developing informal, event-based surveillance, i.e. detection of disease emergence using a broad range of intelligence sources (e.g. social networks, online news reports, scientific articles) [4–7]. For example, following the first introduction of ASF in Lithuania in 2014, massive wild boar mortality and suspicion of ASF was first reported in the electronic media, followed by official confirmation of ASF outbreaks in January 2014 [8].

### French Animal Health Epidemic Intelligence System

In 2011, a joint initiative of multiple French stakeholders in the animal health sector resulted in the launch of the French Animal Health Surveillance Platform (ESA Platform). ESA Platform members collaborate to help improve national epidemiological surveillance, centralisation and dissemination of animal health data.

In 2013, the ESA Platform formed a multidisciplinary team to oversee the Animal Health Epidemic Intelligence System (Veille Sanitaire Internationale, VSI) to ensure the detection, verification and communication of signals of infectious animal diseases that emerge outside France. Current diseases of interest to VSI are African swine fever (ASF), foot-and-mouth disease (FMD), bluetongue (BTV) and avian influenza (AI), including both low pathogenic avian influenza (LPAI) and highly pathogenic avian influenza (HPAI).

Epidemiologists and information technology specialists from the VSI team collect data from indicator- and event-based surveillance systems daily. The team relies on formal notifications from sources such as the World Organisation for Animal Health (OIE) and the European Commission (EC) for the indicator-based component, while it consults webpages from general and animal health news sites, along with biosurveillance systems such as ProMED for the event-based part. Moreover, the team receives confidential information from a network of experts from reference laboratories and regional and international disease surveillance programs, who also verify signals for VSI. For all verified information, the VSI team sends *ad-hoc* reports to members of the ESA Platform, and posts freely available online reports for all officially confirmed outbreaks [9, 10].

Time-consuming manual consultation of numerous news websites is an ongoing impediment for VSI despite its substantial human capacities and organisation. In addition, the biosurveillance systems consulted by VSI principally cover public health topics and are therefore of limited value with regard to the diseases of interest.

Here we present the Platform for Automated extraction of Disease Information from the web (PADI-web), an automated biosurveillance system for collection, processing and extraction of epidemiological information from online news sources. The core component of PADI-web is a combined method for information extraction (IE) founded on rule-based systems and data mining techniques. This combined method generates epidemiological information on diseases, locations, dates, hosts and number of cases for outbreaks mentioned in the news.

We evaluate the accuracy of the IE component of PADI-web to extract epidemiological information from a corpus of news reports. We further assess the ability of PADI-web to detect ASF, FMD, BTV and AI outbreaks that were reported outside France from January to June 2016. We compare our results with two major international biosurveillance systems, one manual and the other semi-automatic.

## Methods

In this section we present the main definitions used in this study and describe how PADI-web collects (step 1), processes (step 2) and extracts (step 3) epidemiological information from online news sources.

### Definitions

Definitions with epidemiological context:

- **Outbreak** is a verified occurrence of an infectious animal disease. A primary outbreak is the first occurrence or re-occurrence of a disease notifiable to the World Organisation for Animal Health (OIE). A primary outbreak is subject to immediate notification to OIE. A secondary outbreak is a spread of a primary outbreak. A secondary outbreak is subject to follow-up notification to OIE. Once an outbreak is resolved, a country sends OIE an immediate notification for closure of that event. For outbreaks of enzootic diseases notifiable to the OIE, countries send OIE 6-monthly reports with summarized quantitative or qualitative data.
- **Epidemiological information** is data on the disease, location, date, host and number of cases from an outbreak.
- **Signal** is an unverified set of raw epidemiological information for an outbreak. It is based on a location, associated with epidemiological entities such as disease, hosts or number of cases (PADI-web evaluation protocol section). Upon verification, the signal can be relevant (related to an outbreak); but otherwise the signal is irrelevant.
- **Disease-related (relevant) news** is news mentioning outbreak(s) or suspicion(s) thereof.

Definitions within the IE context:

- **Information types** are the different entities of epidemiological information that we want to extract from the news. In the current version of PADI-web, there are five information types: diseases, locations, dates, hosts and number of cases.
- **Candidate** is a text fragment of the same type as the desired information type, identified without taking the epidemiological context into account. For instance, when looking for a date of a specific outbreak in the news, all dates mentioned in the news are candidates, even if some of the dates are not related to the outbreak.
- **Candidate class** can be correct or incorrect. If a candidate is the desired information type, then it is correct; if not, then it is incorrect.

### Data collection

Similarly to other biosurveillance systems [11–13], PADI-web collects online news directly from structured data services, i.e. Really Simple Syndication (RSS) feeds. PADI-web currently gathers news via Google News RSS feeds. We opted for Google News in the English language because it is a free news aggregator with global coverage of about 4,500 news sites. Moreover, Google News RSS feeds can be customized using search terms proposed by users.

PADI-web thus implements two types of RSS feed to collect relevant Google News. The first type of feed consists of disease name terms (e.g. “African swine fever”, “warthog disease”). The second type of feed consists of associations of terms describing a clinical sign and host for a given disease (e.g. “wild boar mortality”, “pig mortality” with regard to African swine fever). Through this unique approach, PADI-web is able to detect relevant news for our diseases of interest, as well as other diseases that share the same host and clinical signs as the diseases we are interested in.

Moreover, our list of terms of disease names, hosts and clinical signs is derived from vocabulary describing outbreaks in a set of relevant news reports [14]. To obtain our list of terms, we use BioTex, a tool for automatic extraction of biomedical terms from free text [15]. BioTex extracts terms based on two principles: i) a relevant combination of information retrieval techniques and statistical methods (e.g. TF-IDF, OKAPI and C-value), and ii) a list of syntactic structures linked with the Medical Subject Headings (MeSH) biomedical thesaurus.

For each disease of concern, we first collect a set of about 200 relevant news reports and extract about 2,000 biomedical terms. Next, a veterinary epidemiologist selects the relevant terms, i.e. disease names (e.g. “African swine fever”, “warthog disease”, “ASF”), clinical signs (e.g. “mortality”, “death”, “fever”) and hosts (e.g., “pig”, “wild boar”). A group of five disease specialists evaluates the terms highlighted by the veterinary epidemiologist and selects the most appropriate ones that describe a given disease. The final list includes the terms validated by the majority of specialists [14]. We also use this list of terms in the data processing and IE steps.

### Data processing

PADI-web uses a simple automated method for processing collected news. Initially, our system checks if the news title and headline include terms such as “outbreak” and a disease name or an association between a clinical sign and host (see Data collection step). All unique URLs and contents are considered to be relevant news and are stored in the PADI-web database for the IE step.

### Information extraction

**Fig 1:**
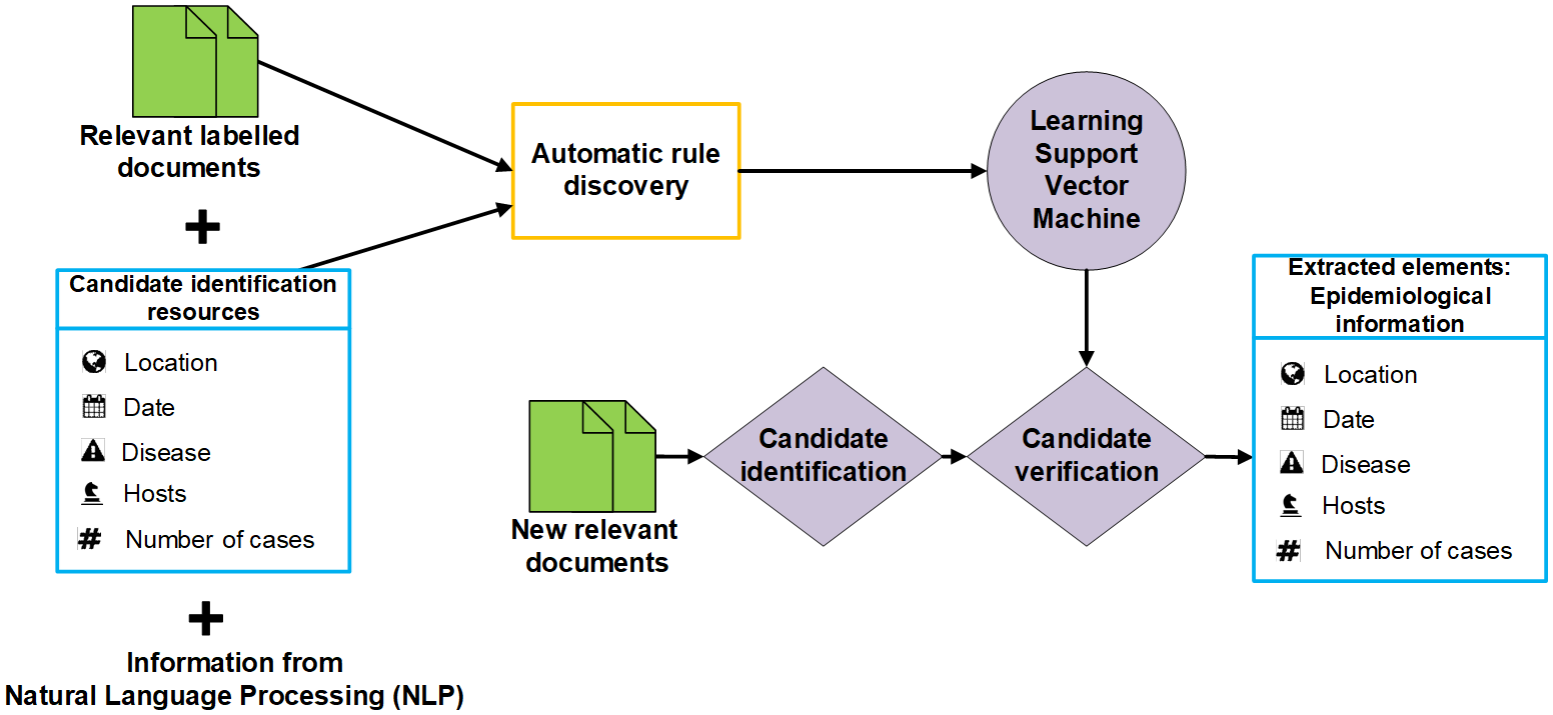
Information extraction step implemented in PADI-web.

Information extraction (IE) is the core component of PADI-web. The aim is to extract epidemiological information for an outbreak described in the news, and ultimately to detect the disease (e.g. “African swine fever”), location (e.g. “Lithuania”, “Alytus county”) and date (e.g. “Friday, Dec 13”, “last Tuesday”) of an outbreak, while also identifying the affected host (e.g. “cattle”, “pig”, “sheep”) and the number of cases (e.g. “15 cases”). IE consists of two stages: i) candidate identification, and ii) candidate verification (Fig 1).

#### Candidate identification

Candidate identification aims to detect all possible candidates for each information type that we want to extract from the news, regardless of the epidemiological context. This is the IE primary filter stage and has the advantage of being compatible with external and generic resources and tools for candidate identification, such as:

- **Diseases and hosts**. Text fragments that match one of the names from the lists previously compiled in the Data collection step [14, 16].
- **Locations**. Text fragments that match location names from the gazetteer GeoNames [17]. To reduce the number of location names from GeoNames, we consider only locations with a population greater than 150,000 (this threshold was selected empirically to ensure that the corresponding number of locations will fit in the main memory), and of type A (countries, regions, states) or P (towns, cities).
- **Dates**. Dates are identified using the rule-based system HeidelTime [18].
- **Number of cases**. Every number in the text is a candidate for the number of cases. We use a list of regular expressions to identify numbers both in numerical or textual form.

The general candidate identification principle is exemplified with the two following sentences (*S*):

- *S*_1_. “12 pigs have been infected by ASF in Poland.”
- *S*_2_. “Authorities in Brussels have declared that the situation is taken seriously.”

In *S*_1_ and *S*_2_, “Poland” and “Brussels” are locations in GeoNames and are therefore candidates for an outbreak location.

#### Candidate verification

In the previous example, “Poland” in *S*_1_ refers to the location of an outbreak (correct candidate), while “Brussels” in *S*_2_ does not. Intuitive rules may be used to decide whether a location candidate refers to an outbreak location, such as:

- *R*_1_: a location found in the same sentence as a host and a disease is probably the outbreak location.
- *R*_2_: a location preceded by a disease and the word “in” is probably an outbreak location.
- *R*_3_: a location found in the same sentence as the verb “declare” is likely not an outbreak location.

To avoid having to manually draw up IE rules, we introduce a method where rules such as *R*_1_, *R*_2_ and *R*_3_ are automatically discovered in a dataset of news where all candidates are pre-labelled as correct or incorrect. We describe this dataset in the Evaluation protocol for information extraction section.

#### Automatic rule discovery

Our automatic rule discovery is based on *frequent itemset discovery*, a widely known data mining technique [19, 20]. This technique can reveal correlations in a large volume of data by providing elements that frequently occur together in the dataset. In order to use the algorithms for *frequent itemset discovery*, we first transform every candidate into a set of elements that describe the candidate and context in which it occurs in the news. We detail how elements that describe each candidate are constructed based on the example in *S*_1_:

- **Elements related to the word**. Each word positioned around a candidate is encoded as an element which describes both the word itself and its position relative to the candidate. For example, the element (infected, -4) means that the word “infected” is four words before the location candidate “Poland”. The position of an element is also expressed according to the relative position of the sentence or the paragraph in which it is found. For example, (infected, -1 sent) reveals that the word “infected” is in the sentence preceding the sentence candidate. We also tailor the definition of the position of an element to make it less precise, e.g. (infected, -1 to -5) means that the word “infected” is one of the five words preceding our candidate.
- **Elements related to word abstraction**. Each word is abstracted with additional information. For example, a word is associated with its grammatical function (e.g. verb) or its lemma (the canonical form of a word; e.g. pig is the lemma of pigs), both produced by TreeTagger [21]. Moreover, the element (disease, -2) means that a disease name is found two positions before the candidate “Poland” and the elements (verb past participle, -4) and (lemma: infect, -4) respectively indicate that a past participle is found four tokens before our candidate, with this verb being “to infect”.
- **Elements related to the position**. We also identify the position of a given candidate in the entire news text. This position is expressed with respect to the paragraph in which this term is located. For example, the element (position, 0%-10%) means that the current candidate occurs in the first 10% of the document, while (position, PAR1) means that it is in the first paragraph.

Each candidate in the labelled dataset is thus associated with a set of elements that describe its context. The automatic rule discovery algorithm is executed for each candidate class. For example, the following rule is automatically discovered in the class which corresponds to the correct number of infected animals: (killed, -1 to -3), (position, PAR1). This rule states that when the word “killed” is found in one of the three words preceding a number (i.e. a candidate for a number of cases) in the first paragraph of the document, then this number likely belongs to the class of correct numbers of cases.

Each rule is also associated with its confidence value, which is the probability that a candidate complying with this rule belongs to its corresponding class. For instance, the previous rule (killed, -1 to -3), (position, PAR1) has a confidence of 83%, which means that 83% of candidates complying with this rule are correct numbers of cases.

This confidence is used to perform an automatic selection process and reduce the number of rules for each class; we only retain rules that have the best confidence among redundant rules (two rules are redundant if they apply to the same candidates in their class).

#### Training a classification model for candidate verification

Once rules are generated for a candidate class, we still need to decide whether a candidate is correct or incorrect. We thus create a description matrix (Table 1) for each class, where candidates are rows and rules are columns. A candidate that satisfies a rule then gets a value 1 and otherwise it gets a value 0.

**Table 1:** Description matrix describing candidates (rows) according to the rules they satisfy (columns). The example shows a description matrix for candidate locations.

Candidate	*R*_1_	*R*_2_	*R*_3_	Class
Poland in *S*_1_	1	1	0	correct
Brussels in *S*_2_	0	0	1	incorrect

We use this description matrix to train a Support Vector Machine (SVM) model with a Radial basis function kernel [22–25]. We train the SVM model on candidates from a labelled dataset described in the Evaluation protocol for information extraction section.

**Location disambiguation**. For location disambiguations, such as the location name “Paris”, which according to GeoNames refers to more than 60 different locations worldwide, we train an additional SVM model to achieve location disambiguation in the news [26]. We train the model based on four features of a given spatial entity, *E*:

- The number of occurrences of *E* in the news report, divided by the total number of spatial entities therein.
- The number of occurrences of spatial entities from the same country as *E* in the news report divided by the total number of spatial entities therein. This feature follows the principle that if *E*’s country is often mentioned in the news then it is more likely to be the correct spatial entity.
- The relative position of *E* in the news (0% when found at the very beginning of the news report, 100% when found at the very end).
- The total number of spatial entities in the given news report.

Each spatial entity is classified as correct or incorrect according to the same principle as in the rule-based classification.

Finally, all candidates that are automatically classified as correct represent the epidemiological information for an outbreak (a signal). This information is available to the VSI team in a structured and tabular format and can be used for further verification and analysis.

### Evaluation protocol for information extraction

We evaluated the accuracy, precision, recall and F-score of the IE method to correctly classify candidates for diseases, dates, locations, hosts and number of cases in a dataset of labelled relevant news.

#### Dataset

Two IE stages require labelled data: i) automatic rule discovery for each candidate class, and ii) training of an SVM model for candidate verification.

We therefore created a labelled dataset for each candidate type (diseases, locations, dates, hosts and number of cases), which we then used for both training and evaluation (S1 Dataset). The dataset consisted of English language news reports collected from the Google News aggregator that mentioned outbreaks notified to OIE from January 2014 to December 2015. We only selected news reports that were published before an OIE notification date. PADI-web is thus trained to find relevant information early, before official notification.

Among the 532 news pieces collected, 352 were evaluated and classified as relevant by a veterinary epidemiologist (EA), i.e. disease-related news. These 352 news reports constituted the training and evaluation dataset. In each of these 352 news reports, the candidates were automatically identified using the method described in the Candidate identification step. A veterinary epidemiologist (EA) and a computer scientist (JR) subsequently labelled these candidates as correct or incorrect regarding their relevance to an outbreak.

#### Metrics

**Accuracy (Acc)** was the proportion of correctly classified candidates by PADI-web over the total number of candidates. **Precision (Pr)** was the proportion of correct candidates over the total number of candidates classified as correct by PADI-web. **Recall (Re)** was the proportion of candidates classified as correct by PADI-web over the total number of correct candidates. **F-score** was the harmonic mean of precision and recall.

We evaluated two IE scenarios: i) candidate identification alone, i.e. how IE performed in cases when all candidates were correct, and excluding candidate verification, and ii) the combined IE candidate identification/verification method.

#### Libraries and tools

IE was implemented in Java 1.8 using existing libraries and tools. The existing jLCM implementation of the LCM algorithm was used in the rule discovery process [20]. Raw text documents were preprocessed using the Readability library (to extract text form the collected news reports), the Stanford CoreNLP tokeniser [27] (to segment the text into a sequence of individual components such as words and punctuation marks) and TreeTagger [21] to generate word abstractions (e.g. lemmas, grammatical functions). The SVM model was built using the LibSVM library [28].

### PADI-web evaluation protocol

We assessed PADI-web regarding the relevance, sensitivity and timeliness in detecting ASF, FMD, BTV and AI signals outside France from 1 January to 30 June 2016. Using the same diseases, evaluation period, geographical coverage and language, we compared PADI-web to two major biosurveillance systems operational today, i.e. ProMED and HealthMap. All signals detected in France were excluded from the analysis since the goal is to monitor disease emergence outside France.

#### Dataset

**PADI-web** is an automatic biosurveillance system that has been operational since January 2016. PADI-web currently collects English language news using the Google News aggregator. The system design and its detailed description are given in the Methods section.

We downloaded the freely available PADI-web dataset (S2 Dataset) from the dedicated webpage [29]. Each row in the PADI-web dataset corresponds to a location candidate extracted from a news report and classified as “correct” (see Information extraction section). Each location candidate is associated with all other correct candidates (diseases, dates, hosts and numbers of cases) extracted from the same news report, and represents a signal (see Definitions section). First, we cleaned this set of signals by removing duplicates and locations having a feature code in GeoNames starting with “PCL” (i.e. political entities, that usually correspond to countries). Such locations were removed because their geographical information scale was considered too broad to reveal any useful knowledge. Second, for each studied disease, we selected signals containing: i) at least one reference to the disease, and ii) one or several hosts specific to the disease.

**ProMED** is a human moderated biosurveillance system that has been operating since 1994. It is the most extensive open-access biosurveillance system and is available in five languages [30, 31]. ProMED’s main source for signals of animal diseases is the OIE, followed by sanitary reports sent from subscribers worldwide. ProMED moderators verify the signals before sharing them with its network of subscribers. Any signals that cannot be verified are tagged with a ‘request for information (RFI)’ [31]. Major signals of international concern are shared in English through ProMED-mail, ProMED’s main network. Specific signals of regional concern are shared with ProMED’s regional networks, e.g. English language posts in ProMED-MBDS (Mekong Basin region of Southeast Asia), ProMED-EAFR (Anglophone Africa), ProMED-MENA (Middle East) and ProMED-SoAs (South Asia).

**HealthMap** is a semi-automatic biosurveillance system that was launched in 2006 [13]. The system automatically collects, processes and displays signals from online sources available in seven languages. HealthMap sources for signals of animal diseases include news aggregators (e.g. Google News, Baidu, Soso, VeriSign), the OIE, as well as national (animal) health authorities and other biosurveillance systems (currently ProMED). Before publishing, HealthMap moderators rate signals on a 1 to 5 scale according to relevance. Signals of minor international concern are rated as 1, while signals of major international concern are rated as 5 [32]. For accurate comparison with PADI-web, we only included HealthMap data obtained from its news aggregators, hereafter referred to as HealthMap (Agg.).

Data from ProMED and HealthMap (Agg.) were freely downloaded from the HealthMap webpage [33]. For each studied disease, one veterinary epidemiologist (EA) filtered the signals reported outside France from January to June 2016. The epidemiologist evaluated each signal by reading the news report content, the location of the event, date and, where relevant, the affected hosts. One news report may consist of one or several signals. A ProMED report sometimes summarizes information on several outbreaks within the same geographical zone or outbreaks subject to the same immediate notification to OIE. Each summarized ProMED report was considered as one signal. One veterinary epidemiologist (EA) cross-checked the ProMED signals in the HealthMap database with reports published on the ProMED webpage [34], and supplemented the dataset if signals were missing. We assessed ProMED using signals from all networks publishing English language reports, i.e. ProMED-mail, ProMED-MBDS, ProMED-EAFR, ProMED-MENA and ProMED-SoAs.

**Confirmed outbreaks** included all data collected from the Emergency prevention system (Empres-i) of the UN Food and Agriculture Organization (FAO) with the same geographical coverage and diseases as included in data collected from PADI-web, ProMED and HealthMap (Agg.). Empres-i centralises data for outbreaks of major diseases of animal and public health concern, collected from OIE (immediate notifications for ongoing and resolved primary outbreaks and follow-up reports for ongoing secondary outbreaks), WHO, national (animal) health authorities, FAO field officers, reference laboratories and research. We downloaded open-access Empres-i data from the Empres-i webpage [35].

The Empres-i dataset does not provide the reasons for outbreak notifications. Therefore, for OIE-sourced Empres-i data, one veterinary epidemiologist (EA) manually matched outbreaks that corresponded to an immediate OIE notification (primary outbreak) or follow-up report (secondary outbreak). All immediate notifications and follow-up reports sent to OIE from January to June 2016 were available in the OIE World Animal Health Disease Information System (WAHID) immediate notification and follow-up report archive [36].

#### Metrics

**Relevance (Rel)** was the percentage of signals detected by PADI-web, ProMED and HealthMap (Agg.) that corresponded to: i) diseases notifiable to OIE where an outbreak was recorded in the Empres-i database during the evaluation period, from January to June 2016 (notifiable, current epizootic category), or before the evaluation period, from January 2014 to December 2015 (notifiable, recent epizootic category), or 6 months after the evaluation period, from July to December 2016 (notifiable, new epizootic category), ii) diseases notifiable to OIE where no outbreaks were recorded in the Empres-i database during the evaluation period, from January to June 2016, and according to the OIE 6-monthly country reports [37] or disease timeline reports, the disease was noted as continuously present or suspected in the country or in one or more of its zones, [38], since at least January 2013 (notifiable, enzootic category), iii) outbreak of another disease, notifiable or not to OIE (other diseases category), iv) disease outbreak awareness, prevention and surveillance (disease vigilance category). If a signal could not be confirmed through the OIE 6-monthly reports or the Empres-i database, and consisted of relevant epidemiological information on the spread or (re-)emergence of a disease, it was categorized as ‘alert’. Signals were considered irrelevant if they did not fit in these categories. Two veterinary epidemiologists (EA and SV) evaluated the relevance of signals based on the content (disease, location, host and date of the events) detected by PADI-web, ProMED and HealthMap (Agg.).

**Sensitivity (Se)** was the percentage of ongoing primary outbreaks from immediate notifications to OIE (hereafter referred to as primary outbreaks) that were recorded in the Empres-i database and which were detected by PADI-web, ProMED and HealthMap (Agg.) (True Positive, TP). False Negatives (FN) were all primary outbreaks from immediate notifications to OIE which were not detected by the evaluated biosurveillance systems.

**Timeliness** was the lag in days from the date of immediate notification to OIE (day 0), as recorded in the Empres-i database, to the date of detection by PADI-web, ProMED and HealthMap (Agg.) during the evaluation period. A negative lag meant that a biosurveillance system was timely in detecting a primary outbreak, i.e. before the date of immediate notification. A positive lag indicated that a biosurveillance system was untimely in detecting a primary outbreak, i.e. after the immediate notification date.

#### Libraries and tools

All statistical computing and evaluations were performed with the R statistical language [39] using existing libraries. Detected signals and notified outbreaks were plotted using the world map of the Natural Earth project (1:50 m resolution) implemented in the R “maps” library [40].

## Results

### Information extraction evaluation

**Fig 2:**
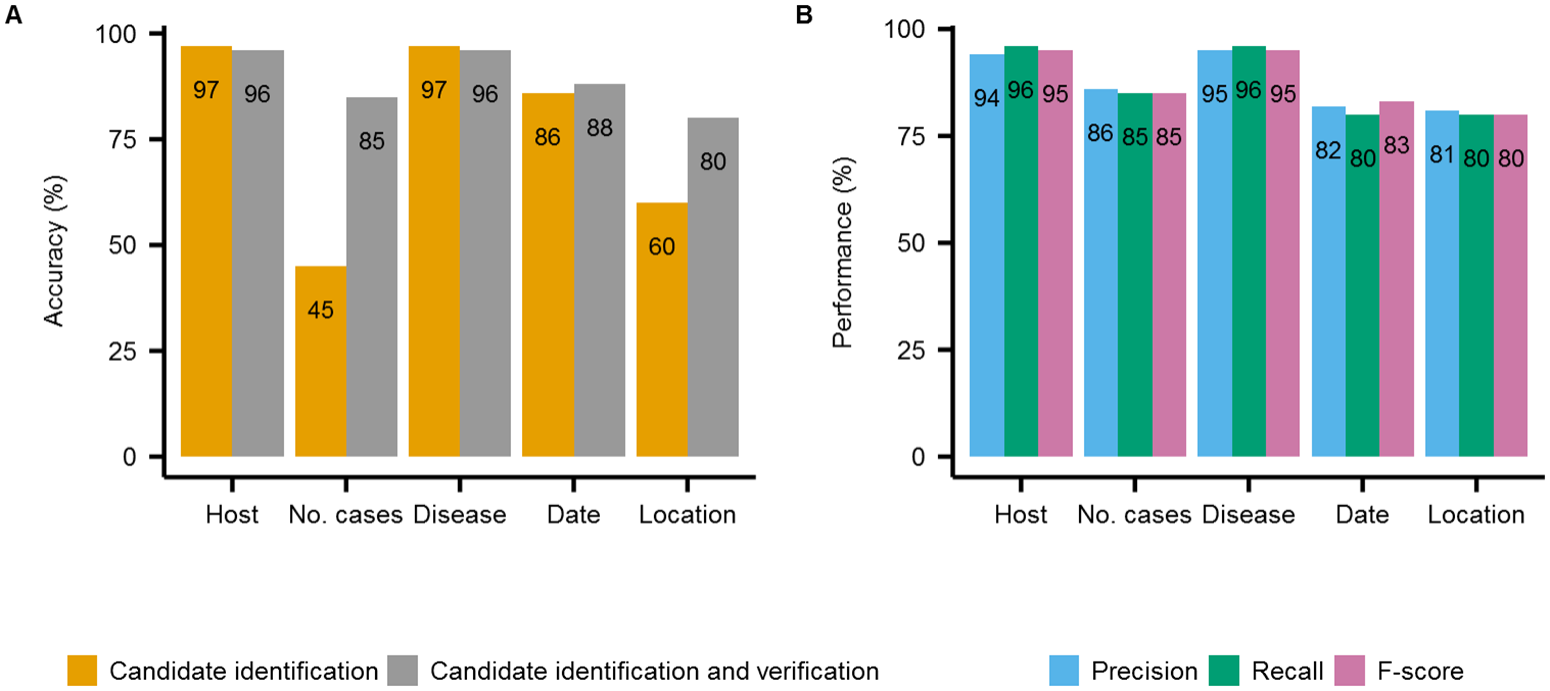
Accuracy of information extraction on a labelled dataset of 352 news reports. A. Comparison of the accuracy (in percentage) of candidate identification alone and of the combined candidate identification/verification based method. B. Precision, recall and F-score (in percentage) of the combined candidate identification/verification based method.

The results of the assessment of our combined IE candidate identification/verification based method, ranged from 80% to 96% (Fig 2). Our results were obtained via tenfold cross-validation on a manually labelled dataset used both for training and evaluation (see Evaluation protocol for information extraction section).

Our combined IE method achieved the highest performance to correctly classify diseases and hosts (F-score of 95%) and lower performance to classify the number of cases, spatial and temporal information types (F-score of 85%, 80%, and 83%, respectively).

The IE accuracy when based only on candidate identification ranged from 45% to 97%. Our combined method for IE was, on average, 30% more accurate than the baseline method which considered all candidates as correct.

These results show that candidate verification is an essential stage for obtaining epidemiological information relevant to PADI-web users.

### Evaluation of PADI-web

#### Confirmed outbreaks

From January to June 2016, Empres-i reported 1,616 ASF, FMD, BTV and AI outbreaks that emerged outside France. OIE contributed 77% (n = 1,240) of the data and national authorities contributed 22% (n = 361). The majority of reported outbreaks concerned ASF in Europe (49%, n = 791) and AI in Africa (24%, n = 381) and Asia (20%, n = 323), respectively. Only 4% (n = 67) were OIE immediate notifications for ongoing (n = 61) and resolved (n = 6) primary outbreaks (Table 2).

**Table 2:** Source and geographical distribution of confirmed outbreaks of African swine fever (ASF), foot-and-mouth disease (FMD), bluetongue (BTV) and avian influenza (AI), reported outside France, from January to June 2016. Percentages for under nine outbreaks are not shown.

Disease Source		Africa	America	Asia	Europe	Total
		n (%)	n (%)	n (%)	n (%)	n (%)
**ASF**	OIE imm notif	6	-	-	5	11 (1%)
	OIE follow-up	3	-	-	786 (49%)	789 (49%)
	**Sub-total**	9 (1%)	-	-	791 (49%)	800 (50%)
**FMD**	OIE imm notif	1	-	15 (1%)	-	16 (1%)
	OIE follow-up	15 (1%)	-	21 (1%)	-	36 (2%)
	Ref laboratory	-	-	1	-	1
	**Sub-total**	16 (1%)	-	37 (2%)	-	53 (3%)
**BTV**	OIE imm notif	1	4	-	3	8
	OIE follow-up	-	-	-	6	6
	**Sub-total**	1	4	-	9	14 (1%)
**AI**	OIE imm notif	2	10 (1%)	16 (1%)	4	32 (2%)
	OIE follow-up	247 (15%)	30 (2%)	64 (4%)	1	342 (21%)
	Nat authority	121 (7%)	-	240 (15%)	-	361 (22%)
	FAO field officer	11 (1%)	-	2	-	13 (1%)
	Ref laboratory	-	-	1	-	1
	**Sub-total**	381 (24%)	40 (2%)	323 (20%)	5	749 (46%)
	**Total**	407 (25%)	44 (3%)	362 (22%)	805 (50%)	1616 (100%)

In January 2016, Georgia submitted an immediate notification to OIE for a first occurrence of BTV in the country. In late January, Georgia submitted a notification to OIE that the event had been resolved, as it was ruled-out that BTV was not the causative agent of the outbreak. Due to the epidemiological importance, we included the alert for the primary BTV outbreak in Georgia in our evaluation of the relevance, sensitivity and timeliness of the three biosurveillance systems.

#### Relevance of signals

From January to June 2016, PADI-web detected 1,065 signals, compared to 158 from ProMED and 361 from HealthMap (Agg.). The majority of signals detected by PADI-web, ProMED and HealthMap (Agg.) were for AI and FMD. Signals for AI accounted for 40% of all signals detected by PADI-web, 60% of those detected by ProMED and 58% of those detected by HealthMap (Agg.). Signals for FMD represented 31% of all signals detected by PADI-web, 36% of those detected by ProMED and 19% of those detected by HealthMap (Agg.) (Table 3).

**Table 3:** Relevance of signals detected by PADI-web, ProMED and HealthMap (Agg.) for African swine fever (ASF), foot-and-mouth disease (FMD), bluetongue (BTV) and avian influenza (AI), from January to June 2016.

Disease Relevance		PADI-web	ProMED	HealthMap Agg.
		n (%)	n (%)	n (%)
**ASF**	Notifiable, epizootic			
	- current	18 (13%)	16 (94%)	63 (79%)
	- recent	2 (1.5%)	-	-
	- new	-	-	3 (4%)
	- alert	1 (1%)	-	9 (11%)
	Notifiable, enzootic	12 (9%)	-	4 (5%)
	Disease vigilance	10 (7.5%)	1 (6%)	1 (1%)
	Other diseases	-	-	-
	Irrelevant	92 (68%)	-	-
	Total	135 (100%)	17 (100%)	80 (100%)
**FMD**	Notifiable, epizootic			
	- current	13 (4%)	12 (24%)	9 (13%)
	- recent	1 (0.4%)	1 (2%)	-
	- new	-	-	-
	- alert	2 (0.6%)	1 (2%)	-
	Notifiable, enzootic	34 (10%)	21 (41%)	54 (79%)
	Disease vigilance	60 (18%)	16 (31%)	3 (4%)
	Other diseases	27 (8%)	-	-
	Irrelevant	198 (59%)	-	2 (3%)
	Total	335 (100%)	51 (100%)	68 (100%)
**BTV**	Notifiable, epizootic			
	- current	4 (2%)	4 (67%)	-
	- recent	4 (2%)	-	-
	- new	-	-	-
	- alert	2 (1%)	2 (33%)	-
	Notifiable, enzootic	2 (1%)	-	-
	Other diseases	14 (9%)	-	-
	Disease vigilance	83 (51%)	-	2 (100%)
	Irrelevant	55 (34%)	-	-
	Total	164 (100%)	6 (100%)	2 (100%)
**AI**	Notifiable, epizootic			
	- current	118 (27%)	69 (82%)	143 (68%)
	- recent	44 (10%)	5 (6%)	2 (1%)
	- new	-	-	-
	- alert	-	3 (4%)	5 (2%)
	Notifiable, enzootic	-	3 (4%)	-
	Other diseases	41 (10%)	-	11 (5%)
	Disease vigilance	83 (18%)	4 (5%)	47 (22%)
	Irrelevant	145 (34%)	-	3 (1%)
	Total	431 (100%)	84 (100%)	211 (100%)

**Fig 3:**
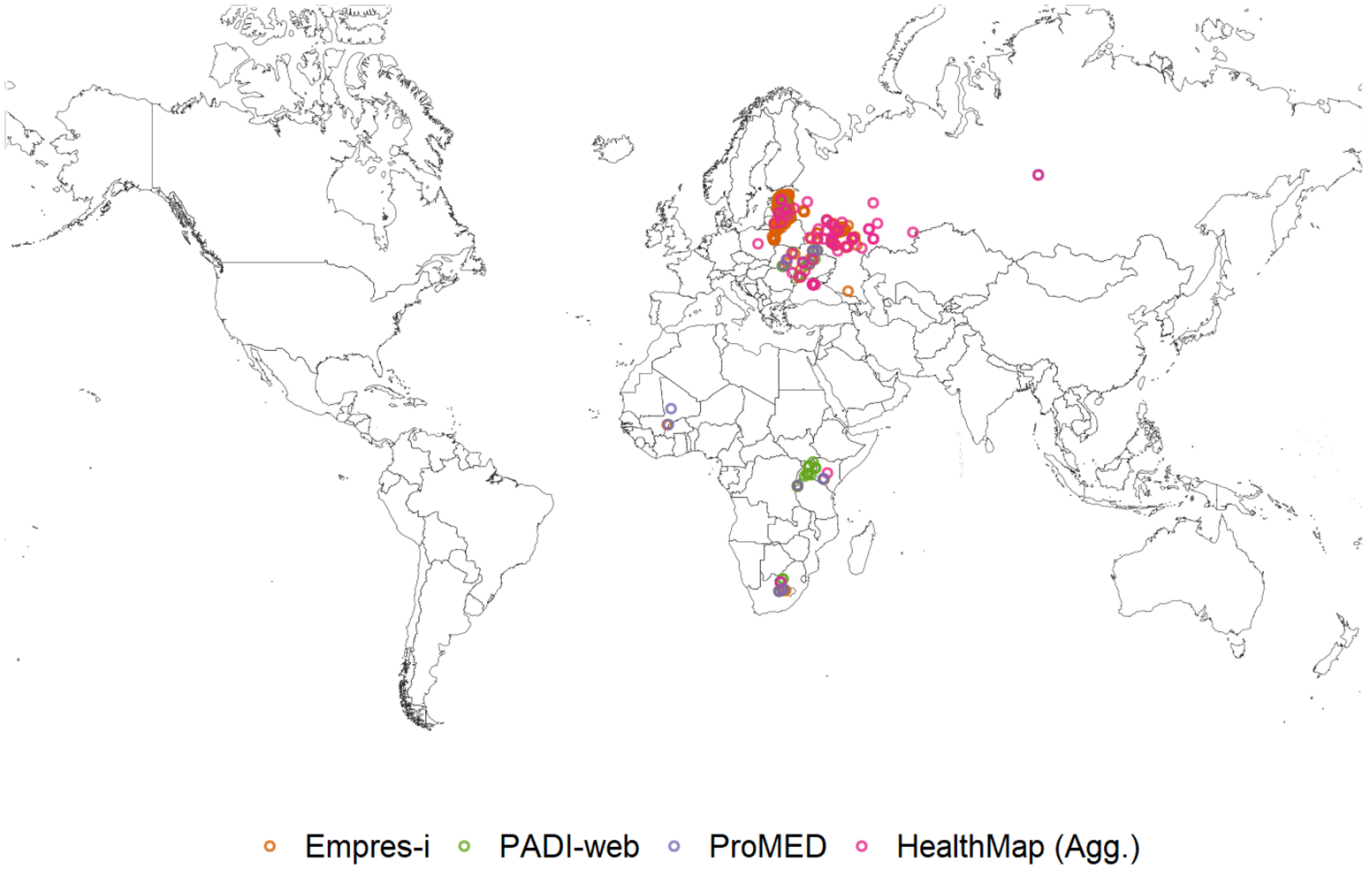
African swine fever (ASF) outbreaks reported in Empres-i and relevant ASF signals detected by PADI-web, ProMED and HealthMap (Agg.) from January to June 2016.

**African swine fever (ASF)**. From January to June 2016, 32% of all signals that PADI-web detected for ASF were relevant to the disease or its current outbreaks, compared to ProMED and HealthMap (Agg.), where all signals for ASF were relevant. The incorrect location of an event was the reason for the 68% of irrelevant signals that PADI-web detected (Table 3, Fig 3).

The majority of signals detected by ProMED and HealthMap were related to current epizootic ASF outbreaks (94% and 79%, respectively), compared to 13% of the PADI-web detected signals that were related to current epizootic ASF outbreaks (Table 3).

Both PADI-web (n = 1) and HealthMap (n = 9) detected signals for ASF outbreaks within an Eastern European zone that we could not confirm via the WAHID 6-monthly country reports and Empres-i databases. In addition, HealthMap (Agg.) reported four signals of vigilance for potential ASF outbreaks within unaffected zones in Eastern Europe, three of which reported primary ASF outbreaks, after our study period, from July to September 2016 (data shown in category new outbreaks) (Table 3, Fig 3).

In African zones where ASF is enzootic, PADI-web detected signals (n = 12) for intensified ASF outbreaks. Similarly, HealthMap, detected signals (n = 4) for intensification of ASF outbreaks in ASF enzootic zones in Eastern Europe. PADI-web also reported signals (n = 10) for ASF preventive measures and vigilance for intensification of outbreaks in epizootically affected countries in Africa and Europe (Table 3, Fig 3).

**Fig 4:**
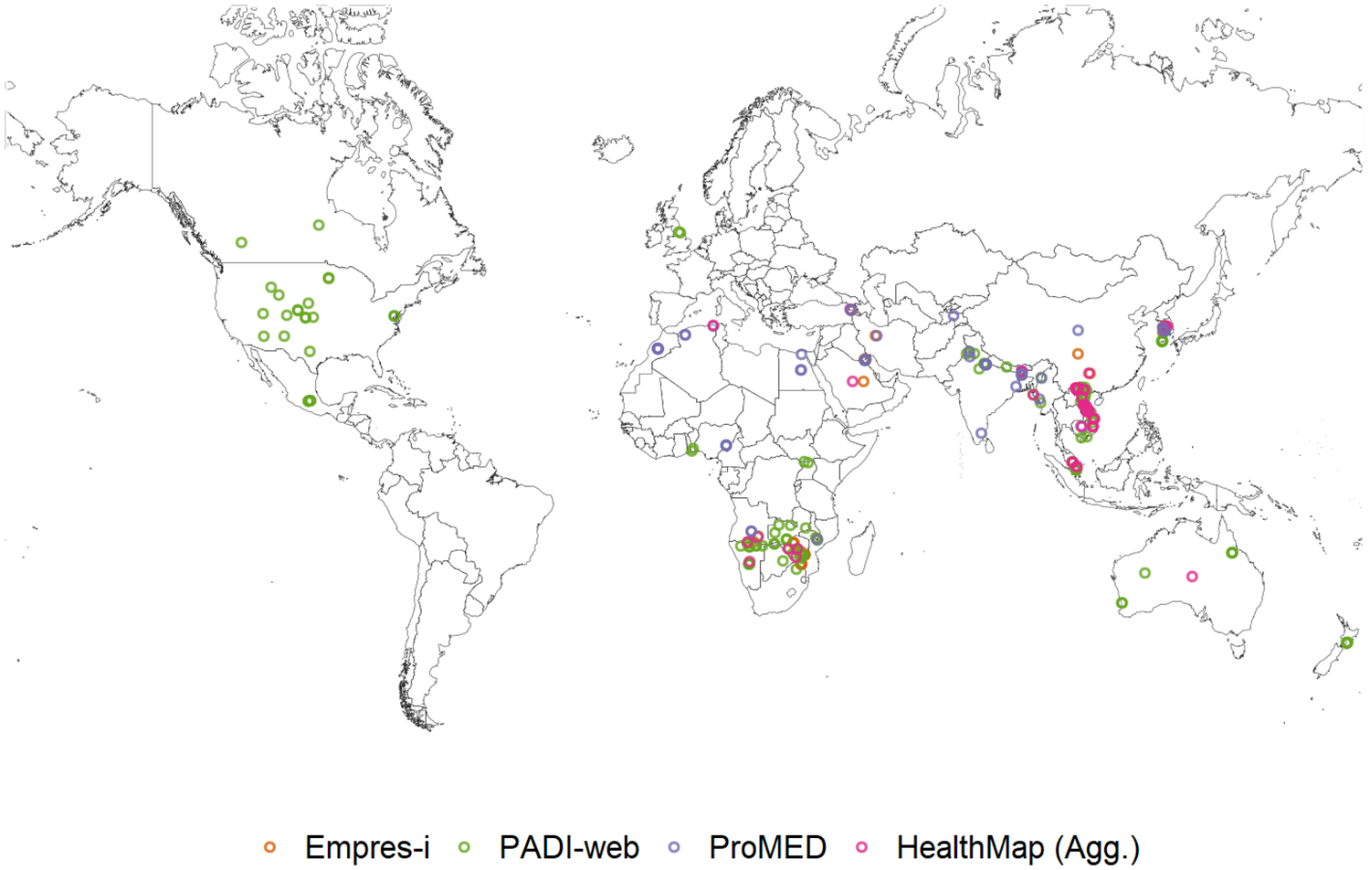
Foot-and-mouth disease (FMD) outbreaks reported in Empres-i and FMD relevant signals detected by PADI-web, ProMED and HealthMap (Agg.) from January to June 2016.

**Foot-and-mouth disease (FMD)**. From January to June 2016, 41% of the signals that PADI-web detected for FMD were relevant to the disease or its current outbreaks, compared to all signals detected by ProMED and 97% of the signals detected by HealthMap (Agg.) that were relevant to FMD. Incorrect location of an event was the reason for 61% of irrelevant signals detected by PADI-web and the two irrelevant signals from HealthMap (Agg.) (Table 3, Fig 4).

The majority of signals from ProMED and HealthMap (Agg.) were related to current epizootic FMD outbreaks (24% and 13%, respectively). In contrast, PADI-web was more exhaustive, detecting signals for current epizootic (4%) and enzootic FMD outbreaks (10%), along with FMD vigilance (18%) (Table 3).

Both PADI-web (n = 2) and ProMED (n = 1) detected signals for FMD outbreaks in zones in Asia that we could not confirm via the WAHID 6-monthly country reports and Empres-i databases. PADI-web also detected signals (n = 27, 8%) for current outbreaks of diseases non-notifiable to OIE (Seneca Valley virus and vesicular stomatitis) in North America and an outbreak of classical swine fever in East Asia, an OIE notifiable disease. Six signals that PADI-web detected for FMD actually concerned cases of hand foot-and-mouth disease in North America (Table 3, Fig 4).

**Fig 5:**
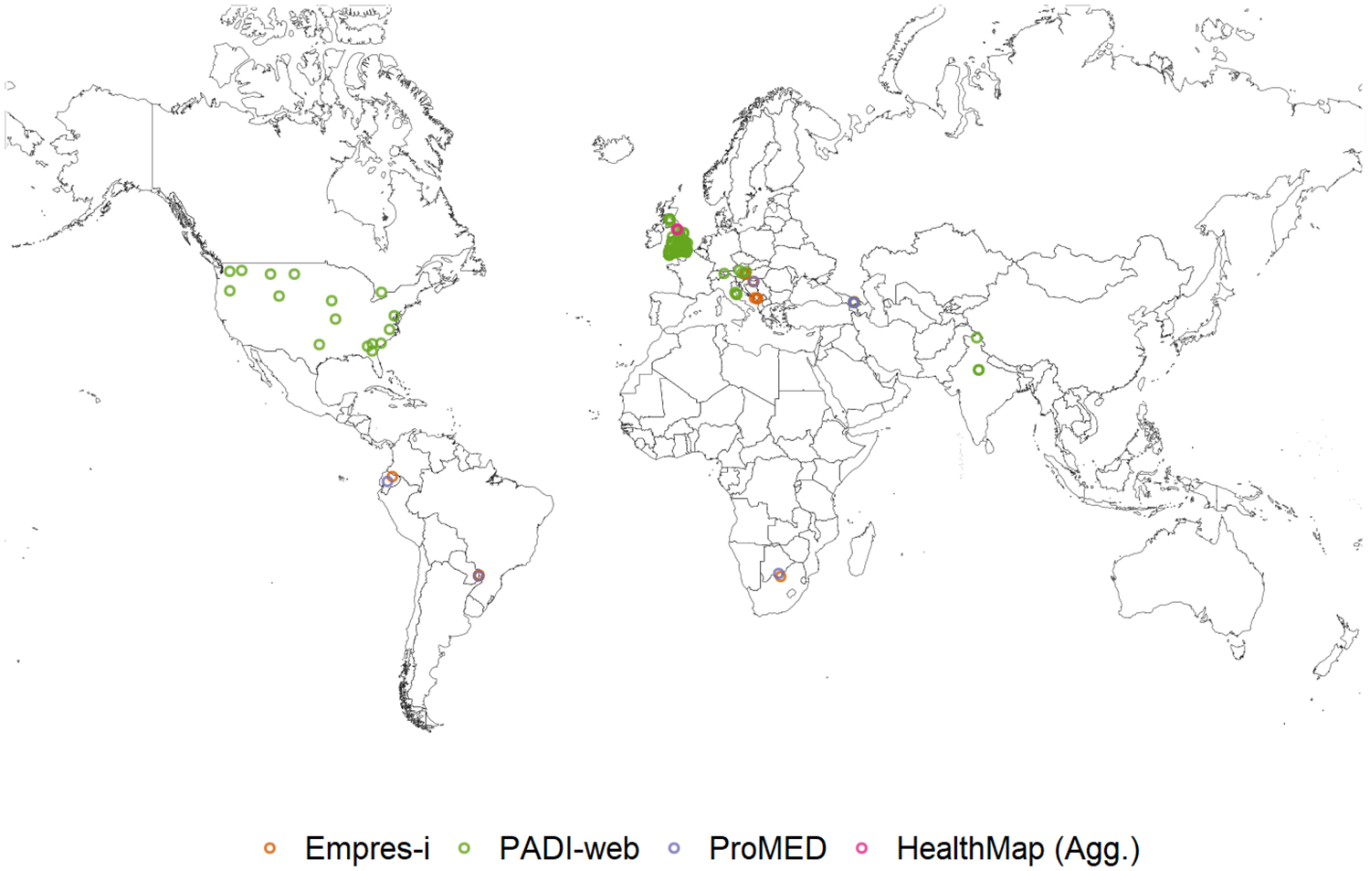
Bluetongue (BTV) outbreaks reported in Empres-i and BTV relevant signals detected by PADI-web, ProMED and HealthMap (Agg.) from January to June 2016.

**Bluetongue (BTV)**. From January to June 2016, PADI-web detected 66% relevant signals for BTV, compared to all BTV relevant signals from ProMED and HealthMap (Agg.). Among the irrelevant signals detected by PADI-web for BTV, 33% had incorrect location of the event and 77% originated from irrelevant news reports (Table 3, Fig 5).

PADI-web was the most exhaustive of all three biosurveillance systems, detecting a variety of relevant BTV signals, whereas ProMED focused on current BTV epizootics (four immediate notifications to OIE and two alerts on BTV in Georgia) and HealthMap (Agg.) reported two signals for BTV vigilance as a result of current BTV outbreaks in Europe (Table 3, Fig 5).

PADI-web also detected signals for current epizootic events in Europe, including two signal alerts for the first occurrence of BTV in Georgia (subsequently ruled-out by national authorities). PADI-web also detected signals for recent BTV outbreaks in North America and signals for the BTV enzooty in Southeast Asian zones. However, the majority of relevant BTV signals from PADI-web were for BTV vigilance, prevention and surveillance in Europe (51%). PADI-web also detected signals (n = 14) for current outbreaks of animal diseases non-notifiable to OIE, adenovirus hemorrhagic disease, Cache Valley virus and chronic wasting disease in North America, as well as malignant catarrhal fever in Europe and North America (Table 3, Fig 5).

**Fig 6:**
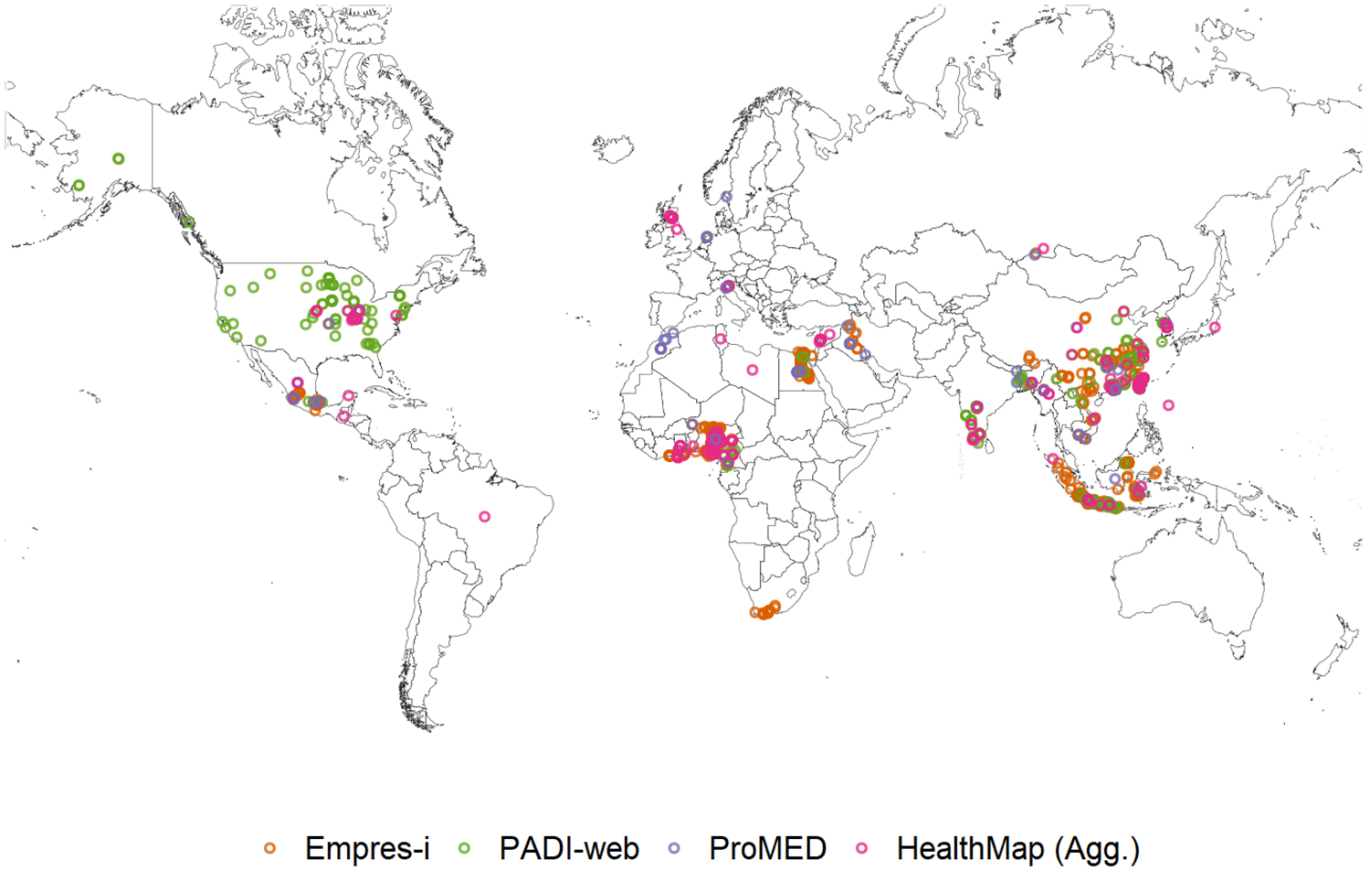
Avian influenza (AI) outbreaks reported in Empres-i and AI relevant signals detected by PADI-web, ProMED and HealthMap (Agg.) from January to June 2016.

**Avian influenza (AI)**. From January to June 2016, 66% of the signals that PADI-web detected for AI were relevant to the disease or its current outbreaks, compared to all signals detected by ProMED and 99% of the signals detected by HealthMap (Agg.). Incorrect location of an event was the reason for 34% irrelevant signals detected by PADI-web and the three irrelevant signals detected by HealthMap (Agg.) (Table 3, Fig 6).

All three biosurveillance systems were exhaustive, covering a range of signals for current, recent and enzootic AI outbreaks, as well as disease vigilance. However, the majority of signals detected by PADI-web, ProMED and HealthMap (Agg.) were related to current epizootic AI outbreaks (27%, 82% and 68%, respectively). ProMED alerted signals (n = 3) on suspicion of primary AI outbreaks in Europe, Africa and Asia. Meanwhile, HealthMap (Agg.) alerted signals (n = 5) on outbreaks in Southeast Asian zones that we were not able to confirm via the WAHID 6-monthly country reports and the Empres-i databases (Table 3, Fig 6).

Both PADI-web and HealthMap (Agg.) reported signals on disease vigilance (n = 83, 18% and n = 47, 22%) for awareness and preparedness for a possible spread of AI due to the current AI outbreaks in North America and Asia. These two biosurveillance systems also reported signals on human cases of AI (n = 41, 10% and n = 11, 5%, respectively)(Table 3, Fig 6).

#### Sensitivity and timeliness of signals

**Fig 7:**
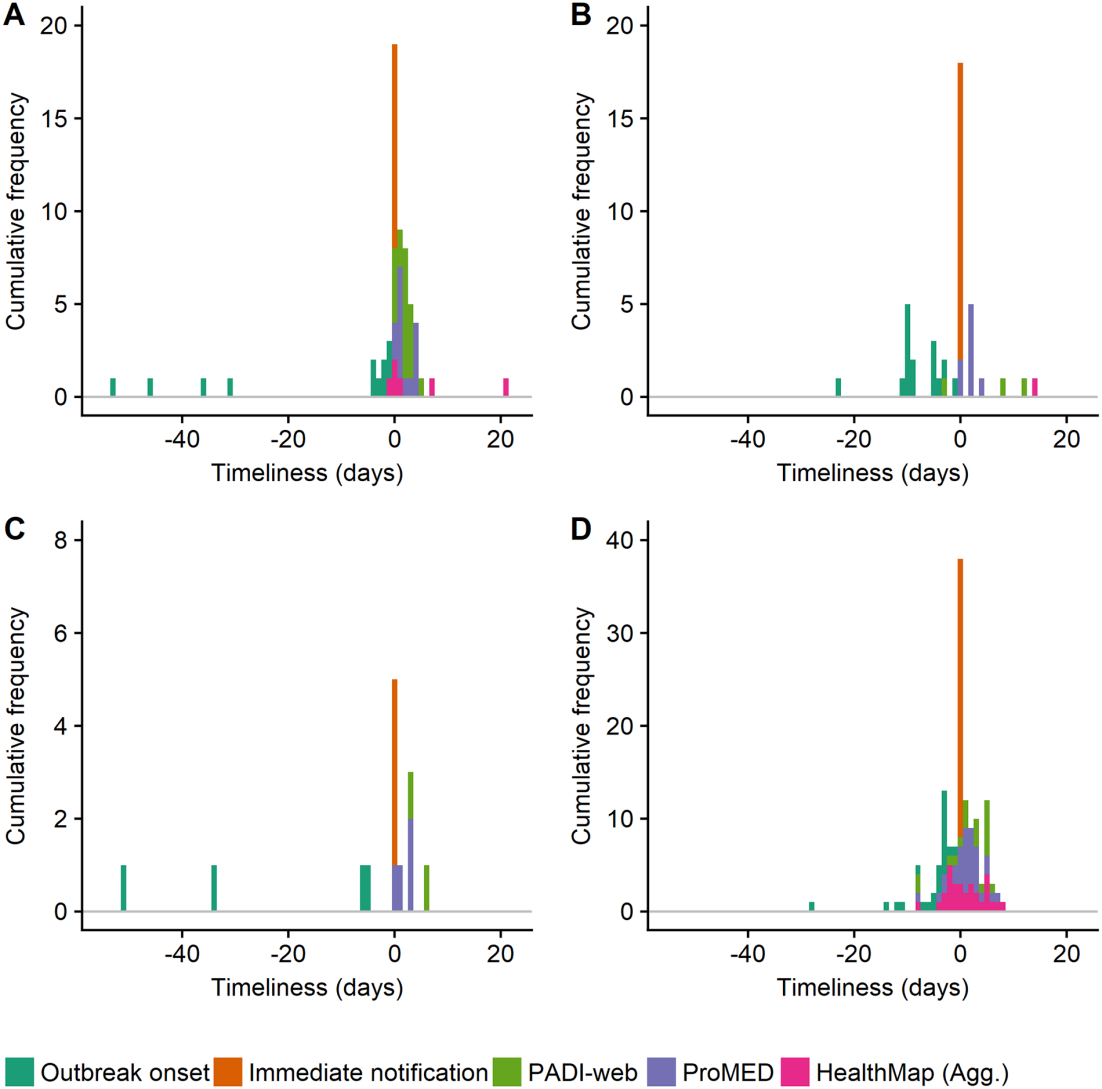
A. African swine fever, B. Foot-and-mouth disease, C. Bluetongue, D. Avian influenza. The figures show the lag in days from the onset of a primary outbreak, its immediate notification to the World Organisation for Animal Health (OIE) and its detection by PADI-web, ProMED and HealthMap (Agg.) from January to June 2016. Zero-day represents the date of immediate notification. The figures show the range from 55 days before to 25 days after immediate notification.

**African swine fever (ASF)**. From January to June 2016, OIE received immediate notifications for 11 primary ASF outbreaks in Ukraine (n = 5), South Africa (n = 2), Burundi (n = 2), Kenya (n = 1) and Mali (n = 1). The majority of primary outbreaks (64%) were notified to OIE within 7 days of their onset. The remaining 36% of primary outbreaks were notified to OIE 30 days after their onset (Fig 7A).

ProMED detected signals for all 11 primary ASF outbreaks, followed by PADI-web (Se = 64%) and HealthMap (Agg.) (Se = 55%) (Table 4). ProMED and PADI-web detected signals for primary ASF outbreaks on average 2 days after notification, compared to HealthMap which detected the signals on average 5 days after notification to OIE (Table 4).

**Table 4:** Sensitivity and timeliness of PADI-web, ProMED and HealthMap (Agg.) in detecting primary outbreaks of African swine fever (ASF), foot-and-mouth disease (FMD), bluetongue (BTV) and avian influenza (AI) from January to June 2016.

Disease	Signals	PADI-web	ProMED	HealthMap (Agg.)
**ASF**	True positive	7	11	6
	False negative	4	0	5
	Sensitivity	64%	100%	55%
	Average delay (days)	2	2	5
	Range of delays (days)	0 to 5	0 to 4	-1 to 21
**FMD**	True positive	3	16	2
	False negative	13	0	14
	Sensitivity	19%	100%	13%
	Average delay (days)	12	2	25
	Range of delays (days)	-3 to 28	0 to 4	14 to 35
**BTV**	True positive	1	4	0
	False negative	3	0	4
	Sensitivity	25%	100%	0
	Average delay (days)	5	2	-
	Range of delays (days)	3 to 6	0 to 3	-
**AI**	True positive	16	30	18
	False negative	14	0	12
	Sensitivity	53%	100%	60%
	Average delay (days)	2	-6	1
	Range of delays (days)	-8 to 6	-90 to 7	-8 to 8

Overall, 22% of the PADI-web signals detected the primary ASF outbreaks on the day of notification to OIE, compared to 14% and 33% of the signals detected by ProMED and HealthMap (Agg), respectively. HealthMap (Agg.) was both the timeliest in detecting one primary ASF outbreak in Eastern Europe one day before notification, and the least timely in detecting another primary ASF outbreak in Eastern Europe 21 days after notification to OIE (Table 4, Fig 7A).

**Foot-and-mouth disease (FMD)**. From January to June 2016, OIE received immediate notifications for 16 primary FMD outbreaks in Armenia (n = 1), Angola (n = 1), Iran (n = 1), Kuwait (n = 12) and the Republic of Korea (n = 1). Primary outbreaks were notified from 1 to 98 days after their onset, with 38% being notified within 7 days of their onset and only one outbreak 30 days after its onset (Fig 7B).

Among the three biosurveillance systems, ProMED detected signals for all primary FMD outbreaks with the earliest signals being detected on the day of notification to OIE.

PADI-web did not detect the primary FMD outbreaks in Kuwait and Iran, thus reducing its sensitivity (Se = 19%). However, PADI-web was the most proactive by detecting the earliest signal for a primary FMD outbreak in East Asia 3 days before notification to OIE. HealthMap (Agg.) detected signals for primary FMD outbreaks in Angola and Armenia (Se = 13%), 14 and 35 days after notification to OIE, respectively. These lags for HealthMap (Agg.) were not surprising as the main focus of the news reports was preventive measures against the spread of FMD outbreaks in Angola and Armenia (Table 4, Fig 7B).

**Bluetongue (BTV)**. From January to June 2016, OIE received immediate notifications for 3 primary BTV outbreaks in Croatia (n = 1), Botswana (n = 1) and Ecuador (n = 1). OIE also received one immediate notification for BTV in Georgia, this was subsequently ruled out in January 2016. All BTV primary outbreaks were notified from 5 to 51 days after their onset, with only two outbreaks notified within 7 days of their onset (Fig 7C).

ProMED detected signals for all primary BTV outbreaks, including the notification from Georgia, with the earliest signal being reported on the day of notification to OIE. PADI-web detected two signals for the primary BTV outbreak in Georgia (Se = 25%), with the earliest signal being detected 3 days after the outbreak was notified to OIE. HealthMap (Agg.) did not detect any BTV outbreak signals (Table 4, Fig 7C).

**Avian influenza (AI)**. From January to June 2016, OIE received immediate notifications for 30 primary AI outbreaks in Bangladesh (n = 1), Cambodia (n = 1), Cameroon (n = 1), Hong Kong Special Administrative Region (SAR) (n = 1), India (n = 2), Iraq (n = 6), Italy (n = 1), Lebanon (n = 1), Myanmar (n = 1), Netherlands (n = 1), Niger (n = 1), Republic of Korea (n = 1), Russian Federation (n = 1), United Kingdom (n = 1) and the United States of America (n = 10). The earliest primary AI outbreak was notified 1 day after its onset, while the latest was notified 131 days after onset. Overall 60% of all primary AI outbreaks were notified within 7 days of their onset, and 23% of all primary outbreaks were notified 30 days after their onset (Fig 7D).

PADI-web detected signals for 16 primary AI outbreaks (Se = 53%) from 8 days before notification and up to 6 days after notification to OIE. Overall, 21% of all PADI-web signals for primary AI outbreaks were detected before notification and 5% on the day of notification to OIE (Table 4, Fig 7D).

ProMED detected signals for all primary AI outbreaks, with the earliest signal being reported 90 days before notification and the latest 7 days after notification to OIE. Overall, 25% of all ProMED signals for primary AI outbreaks were reported before notification and 11% on the day of notification to OIE (Table 4, Fig 7D).

HealthMap (Agg.) detected signals for 18 primary AI outbreaks (Se = 60%), with the earliest signal being detected 8 days before notification and the latest 8 days after notification to OIE. Overall, 40% of the HealthMap signals for AI detected primary AI outbreaks before notification and 10% on the day of notification to OIE (Table 4, Fig 7D).

## Discussion and conclusion

In this paper, we presented the Platform for Automated extraction of Disease Information from the web (PADI-web), a web-based animal health biosurveillance system designed to monitor the worldwide emergence of new and exotic animal infectious diseases. We further described the method used in PADI-web to automatically collect, process and extract epidemiological information from online news sources. Information extraction is a key step of PADI-web and it combines candidate identification/verification to identify epidemiological information in news reports.

### Key information extraction features

The IE method that we implement in PADI-web offers several key features. We obtain relevant results regarding accuracy and F-score using a unique IE approach applicable to different information types (diseases, locations, dates, hosts and the number of cases). While some methods are specially designed and fine-tuned for a given information type, our IE approach is flexible and can cover multiple information types. Moreover, our IE approach has prospects for further development, e.g. integration of new information types and multiple languages.

We also show that the IE efficiency depends on the information type that we want to extract from the news. For example, the extraction of diseases and hosts is equally efficient with the candidate identification based method alone and the combined candidate identification/verification based method (F-score of 95%). The reason for similar results obtained by the two methods is twofold. First, our lists of disease and host names (Data collection step) are accurate for identifying candidates that have a high probability of being the right ones. Second, candidates for diseases and hosts are less likely to be ambiguous terms compared to other candidates, such as location and date candidates.

Compared to candidate identification based IE alone, the combination of the rule-based system HeidelTime and candidate verification slightly improves the accuracy of date extraction (increases from 86% to 88%) while achieving an F-score of 83%. Other rule-based systems for extraction of temporal entities obtain results within the same range, such as HeidelTime [18], which achieves 85% accuracy and an F-score of 86%, SUTime [41] which achieves an F-score of 90% and TimeText [42] which correctly extracts 84% of all temporal entities from free text documents.

Compared to candidate identification based IE alone, our combined IE method significantly improves the accuracy of location extraction (increases from 60% to 80%) and achieves an F-score of 80%. Our results are similar to those obtained in previous studies that used machine learning algorithms to extract locations from news reports, such as the CRF algorithm (F-score of 86%) [18] and neural networks (F-score of 64%) [43]. These results should, however, be interpreted with caution. While traditional methods use simple recognition of locations based on mentions in texts, the IE problem that we address in PADI-web differs in one essential aspect. In PADI-web, we first recognise mentions of locations and then determine whether a given location is related to an outbreak, and we address the location disambiguation. In our future studies, however, we intend to enhance our current classification model for location disambiguation with more complex features.

Finally, the combined IE method, compared to candidate identification alone, significantly improves the accuracy of extraction of the number cases (increases from 45% to 85%) and achieves an F-score of 85%. As each number (in numerical or textual form) is a potential candidate for being the number of cases, the candidate identification stage alone leads to poor results, with only 45% of extracted numbers found to be the real number of cases. The candidate verification stage therefore improves this outcome. We intend to refine automatic rule discovery as part of the candidate verification stage to improve the accuracy of extraction of the number of cases. We also intend to enlarge the corpus of labelled data so that automatic rule discovery will encounter more examples of correct and incorrect candidates, and we will be able to draw up better rules to distinguish them.

Overall, our results show that the IE efficiency varies according to the information type that we want to extract from the news. For candidates such as hosts, diseases and dates, both candidate identification alone and the combined candidate identification/verification based method produce similar results. For some information types, candidates are almost always correct (despite the method used). For instance, in a given news report, it is very uncommon that a disease name is mentioned without being related to an outbreak. On the contrary, for information types that allow more errors (such as the number of cases and locations), the context is necessary to be able to distinguish correct from incorrect candidates, so here the candidate verification stage is playing its full role.

### What type of new knowledge does PADI-web identify?

PADI-web is an exhaustive biosurveillance system. It enables the discovery of four types of new knowledge:

- Emergence of epizootic diseases. PADI-web successfully identified signals for current outbreaks of diseases that are notifiable to OIE and thus of major international importance. Moreover, PADI-web achieved timely detection, ahead of the date of immediate notification to OIE primary FMD and AI outbreaks in East Asia, thus raising awareness of possible disease spread and prevention. PADI-web also provided alerts of ASF, FMD and BTV emergence within new zones, for which we could not find any official confirmation, thus highlighting PADI-web’s potential as an early warning tool.
- Dynamics of enzootic diseases. PADI-web detected signals of possible re-emergence and intensification of outbreaks in countries where our studied diseases were enzootic, such as ASF and FMD outbreaks in Africa and Asia. These signals can provide risk managers with relevant sanitary information, especially regarding disease dynamics and eventual improvement of disease surveillance and eradication policies.
- Emergence of other diseases that are non-notifiable to OIE. The specific combination of key terms that PADI-web uses to detect online news allows the detection of signals for diseases that have clinical signs similar to those we want to study. This original approach allows PADI-web to detect potential emergence of other diseases. For example, PADI-web detected signals for outbreaks of Cache Valley virus, chronic wasting disease in North America and signals for vigilance for Schmallenberg infections in Europe which have clinical signs similar to those of BTV. PADI-web also detected signals for human cases of AI in Asia in people who were in close contact with birds. PADI-web could develop into a ‘One health’ monitoring biosurveillance system thanks to this zoonotic disease detection feature.
- Disease vigilance. Among the relevant signals that PADI-web detected, 7.5% of the signals for ASF, 18% for FMD, 51% for BTV and 18% for AI were for disease awareness, outbreak preparedness, surveillance and preventive measures. These signals give risk managers a broader vision of the potential geographical scope of disease spread, thus enabling them to take early mitigation measures.

### Current limitations of PADI-web

PADI-web currently detects a substantial number of false positive signals. Based on our definition, each single location extracted from the news and labelled as correct creates a PADI-web signal. This approach allows detection of all potentially relevant locations. However, it leads to numerous false positive signals due to: i) multi-references to the same location in the same article, but not identified as duplicates (e.g. references to the same place at different levels of granularity), ii) reference to an irrelevant location regarding a current outbreak (e.g. reference to a former outbreak in another geographical zone). As this was the reason underlying most of the irrelevant signals, we aim to improve the quality of our signal dataset by identifying the nested locations contained in each report, as well as by removing the irrelevant locations.

Compared to ProMED, both HealthMap and PADI-web had lower sensitivity and timeliness in detecting signals for primary outbreaks of emerging diseases notifiable to OIE. On contrary, ProMED’s main source of information is the OIE, thus achieving the highest sensitivity for the primary outbreaks reported to OIE. These results illustrate the varying ability of biosurveillance systems to adequately detect, efficiently process and communicate disease emergence signals. Non-moderated biosurveillance systems, such as PADI-web and partially HealthMap, search the web and detect information in an unbiased manner, but with a risk of getting false positive signals. Compared to PADI-web, which uses a simple filtering method to categorize collected news reports, HealthMap implements an algorithm for automatic classification of collected news, and both HealthMap and ProMED have human verification before online publication of signals.

One interesting aspect to consider with regard to PADI-web is that, while IE assessment alone provides satisfactory results, the overall performance of PADI-web is lower. The IE step has been trained and evaluated in ideal conditions, using only relevant data, while PADI-web handles real-time news streams. PADI-web combines the data collection, data processing and IE steps to obtain the final results, so PADI-web performs in more challenging conditions. Errors that occur at each step of the PADI-web method impact its overall performance.

For example, in real-time conditions, errors that occur in the IE step cannot be corrected if PADI-web collects irrelevant news and the Data processing step does not efficiently filter out irrelevant news. Similarly, PADI-web’s efficiency is negatively affected if it identifies relevant news but the IE process fails to extract epidemiological information. We are thus currently evaluating an SVM model as part of the Data processing step. We intend to tap the labelled dataset used for candidate verification to train the SVM model to filter news more efficiently.

Additional factors that may have influenced the performance of PADI-web were the language and scope of geographical coverage of the news providers [44]. PADI-web currently collects English language news from Google News. In comparison, HealthMap has access to several news aggregators in seven languages, and ProMED gets first-hand information from subscribers from around the world and its regional networks. Other automated biosurveillance systems, such as BioCaster have achieved high performance for given regions, sources and languages, such as the Asia-Pacific region [45]. The MedISys system favors broader coverage of sources in more than 40 languages but its efficiency has yet to be evaluated [46]. For the future improvements of PADI-web we intend to take these geographical and language factors into account.

Finally, the results of our evaluations revealed some of the limitations of conventional indicator-based surveillance systems, especially regarding lags in official reporting [47]. In average 43% of the primary outbreaks that we analysed in this study were notified to OIE 1 week after their onset and 27% after 30 days of their onset. These lags may have major impacts such as rapid spread into uninfected territories such as the spread of ASF in Eastern Europe in 2007 [3]. Our findings highlighted the necessity for timelier outbreak notification and evaluation of factors that influence official reporting [32, 47, 48].

### Conclusion

In conclusion, PADI-web incorporates intelligent systems based on natural language processing, machine learning and data mining techniques. Our contribution enhances the state-of-the-art of epidemic intelligence and biosurveillance systems. PADI-web has the means necessary to ensure easy monitoring of online news for multiple animal infectious diseases of importance to risk evaluators and risk managers.

## Supporting information

S1 Dataset PADI-web corpus: News manually labelled. Dataset of news reports in English language used to evaluate and train the information extraction module of PADI-web (http://epia.clermont.inra.fr/vsi). This dataset is composed of 532 news reports (in JSON), with information about the report itself (publication date, title, content, url), as well as processing information related to the information extraction process (candidates for extraction information, correct or incorrect labels for each candidate). The named entity candidates (locations, diseases, hosts, dates, number of cases) are manually labelled in each article. Data are shared under a CC BY license and are freely available for download at: https://dataverse.cirad.fr/dataset.xhtml?persistentId=doi:10.18167/DVN1/KMTIFG.
(GZ) Click here for additional data file.

S2 Dataset PADI-web dataset manually evaluated. Dataset of manually evaluated and labelled for relevance signals detected by PADI-web (http://epia.clermont.inra.fr/vsi) from January 1st to June 30th 2016, for African swine fever (ASF), foot-and mouth disease (FMD), bluetongue (BTV) and avian influenza (AI). Each row represents the extracted epidemiological information about a potential outbreak (signal) from the collected news. Each signal is constructed with one location automatically detected as correct in the news, which is combined with all other epidemiological information types that are detected in the same news article. Data are shared under a CC BY license and are freely available for download at: https://dataverse.cirad.fr/dataset.xhtml?persistentId=doi:10.18167/DVN1/JZM34U.
(XLS)
